# Comparison of Multimodal Large Language Models and Oral and Maxillofacial Radiologists in the Detection of Incidental Findings on Panoramic Radiographs: A CBCT-Referenced Diagnostic Accuracy Study

**DOI:** 10.3390/healthcare14182948

**Published:** 2026-09-10

**Authors:** İsmail Çapar, Utku Cem Hasırcı, Didem Dumanlı Kusay, Edanur Altın, Gediz Geduk

**Affiliations:** Department of Oral and Maxillofacial Radiology, Faculty of Dentistry, Zonguldak Bülent Ecevit University, Zonguldak 67100, Turkeydidem.dumanli@hotmail.com (D.D.K.); gedizgeduk@gmail.com (G.G.)

**Keywords:** incidental findings, multimodal large language model, artificial intelligence, panoramic radiography, radiology

## Abstract

**Highlights:**

**What are the main findings?**

- In a finding-enriched dataset, two expert oral and maxillofacial radiologists achieved higher overall diagnostic performance than three multimodal (image-capable) LLMs in detecting nine incidental findings on panoramic radiographs against a CBCT-based reference standard (sensitivity 89.5–91.6% vs. 75.1–83.1%).
- The gap was widest in the exploratory high-risk group (expert sensitivity above 90% vs. 62.9–78.0% for the LLMs); at the level of individual findings, the difference remained significant after correction for multiple comparisons only for carotid artery calcification and extensive maxillary sinus pathology.

**What are the implications of the main findings?**

- Under the consumer-interface conditions and access period tested, general-purpose multimodal LLMs should not be relied on independently to evaluate panoramic radiographs for incidental findings, particularly high-risk ones.
- Any future role for these models is more likely to be as an expert-supervised adjunct or screening aid than as a replacement for expert interpretation; such a combined workflow was not tested here and would require prospective evaluation in clinically representative datasets.

**Abstract:**

**Background/Objectives:** This study aimed to compare the diagnostic performance of multimodal (image-capable) large language models (LLMs) and oral and maxillofacial radiologists in detecting nine predefined incidental findings on panoramic radiographs, using a cone-beam computed tomography (CBCT)-derived reference standard. **Methods:** This retrospective diagnostic performance study included 500 purposively assembled, finding-enriched panoramic radiographs paired with CBCT images. CBCT images were evaluated by three radiologists to establish the reference standard. Two experts and three LLMs, the latter accessed through their consumer web interfaces, independently assessed the presence or absence of the nine findings; 4500 finding-level decisions were analyzed for each reader. Sensitivity, specificity, accuracy, and error rates were calculated. Within-patient clustering was accounted for using generalized estimating equations and a cluster bootstrap procedure with 5000 resamples. Finding-specific comparisons used Cochran’s Q test with Benjamini–Hochberg correction. **Results:** Agreement between the two experts was very good (κ = 0.86). Expert sensitivity, specificity, and accuracy ranged from 89.5 to 91.6%, 92.0–93.0%, and 91.8–92.9%, respectively, compared with 75.1–83.1%, 88.0–90.0%, and 86.8–89.0% for the LLMs. The overall reader effect was significant for all three performance metrics (all *p* < 0.001). In the exploratory high-risk group, expert sensitivity ranged from 90.9 to 93.2% versus 62.9–78.0% for the LLMs. After correction for multiple comparisons, the difference between readers remained significant only for carotid artery calcification (q < 0.001) and extensive maxillary sinus pathology (q = 0.005). **Conclusions:** Under the consumer-interface conditions and access period tested, the LLMs performed below the experts, particularly for high-risk findings, and should not be used independently to evaluate panoramic radiographs. Because the dataset was finding-enriched and single-center, the absolute estimates cannot be transferred directly to routine clinical populations, and any future role for these models, more plausibly as an expert-supervised adjunct or screening aid than as a replacement for expert interpretation, remains to be tested prospectively.

## 1. Introduction

Panoramic radiography is widely used in dental practice because it offers a broad view of the maxillofacial region at low cost and with easy accessibility. Beyond assessing the primary imaging indication, it can also reveal incidental findings such as carotid artery calcifications, maxillary sinus pathologies, tonsilloliths, and sialoliths [1]. Cone-beam computed tomography (CBCT) studies have likewise reported incidental findings at varying prevalences across the maxillofacial region, some of which warrant further evaluation or a change in clinical management [2,3]. Systematic examination of the entire imaged field therefore matters for more than dental diagnosis alone: it also bears on patient safety and appropriate clinical referral.

The clinical significance of incidental findings ranges from benign changes requiring no follow-up to conditions that warrant additional imaging, treatment planning, or referral to the relevant specialist [4,5]. Some findings matter beyond the dental region itself. Carotid artery calcifications visible on panoramic radiographs, for instance, should be evaluated carefully because of their possible association with an increased risk of cerebrovascular disease [6], and CBCT-based studies have reported similar associations between vascular calcifications and systemic risk factors such as hypertension, cardiovascular disease, dyslipidemia, diabetes mellitus, and advanced age [7]. Accurate recognition of incidental findings can therefore support dental diagnosis and treatment planning while also helping to flag possible systemic health risks and prompt appropriate referral for care.

The two-dimensional nature of panoramic radiography, however, imposes real limitations on the evaluation of incidental findings. Anatomical superimposition, geometric distortion, variable magnification, and positioning-related differences in image quality can make findings harder to detect, particularly in small or anatomically complex regions [8,9,10]. A systematic image-review approach and observer experience are therefore among the key determinants of diagnostic performance, and CBCT can serve as a complementary method for the three-dimensional assessment of equivocal or uncertain findings identified on panoramic images [8,11,12].

Artificial intelligence-based image analysis systems have accordingly drawn growing interest for their potential to support diagnostic workflows in dental and maxillofacial radiology [13,14,15]. Deep learning algorithms, in particular, have shown promising performance on specific tasks such as detecting and classifying dental, periapical, and osseous pathologies on two-dimensional dental radiographs [15,16,17]. Most of these systems, however, are task-specific algorithms built around a single pathology, which can limit their usefulness when several findings, spread across different anatomical regions and varying in radiographic appearance, need to be assessed together on the same image.

The emergence of multimodal large language models (LLMs), able to process image as well as text input, has opened new possibilities for evaluating radiological images [18,19], although their reliability for direct image interpretation and diagnostic decision-making remains uncertain. LLM performance in lesion detection, image classification, and complex visual interpretation has been reported to vary with the model and the finding being assessed [19,20,21], and dental radiology studies on panoramic radiographs point to a similar pattern [22]. This variability is likely to be more pronounced when several incidental findings, differing in visibility, anatomical location, and clinical implication, are evaluated on the same image, which is why LLM performance needs to be weighed against an appropriate reference standard and against human experts before these models can be used for clinical decision support.

The number of studies on LLMs in dental and maxillofacial radiology is growing, but the existing literature has focused mainly on automated reporting, educational applications, recognition of specific dental findings, or narrowly defined diagnostic tasks [23,24,25]. To our knowledge, no study has yet compared LLMs with expert radiologists on the detection of multiple incidental findings spanning different anatomical regions on panoramic radiographs, or examined performance by finding category. A recent systematic review, moreover, found considerable variability across studies in the diagnostic accuracy, consistency, and reference-standard agreement of LLMs [23], a gap that calls for evaluating not just their potential role in clinical decision support, but also the patient-safety implications of false-negative and false-positive results.

This study set out to compare the diagnostic performance of multimodal (image-capable) LLMs and oral and maxillofacial radiologists in detecting nine predefined incidental findings on panoramic radiographs, using a reference standard based on CBCT examinations and expert consensus. Secondary, exploratory analyses examined performance differences across anatomical finding categories and predefined clinical risk levels. The aim of this comparative approach was to clarify the current limitations of these models in panoramic radiograph evaluation and to inform the design of future studies of their expert-supervised use.

## 2. Materials and Methods

### 2.1. Study Design

This retrospective diagnostic performance study evaluated panoramic radiographs obtained from patients who presented to the Department of Oral and Maxillofacial Radiology of a university’s Faculty of Dentistry between January 2021 and December 2025. Cone-beam computed tomography (CBCT) images served as the reference imaging modality for confirming the predefined incidental findings assessed on the panoramic radiographs. The study was reported in accordance with the STARD 2015 guideline for diagnostic accuracy studies and the CLAIM 2024 update for imaging studies involving artificial intelligence; the completed checklists are provided in Supplementary File S1 and Supplementary File S2, respectively.

### 2.2. Ethical Approval

Ethical approval for the study was obtained from the Non-Interventional Clinical Research Ethics Committee (Approval No: 2026/08). All procedures were conducted in accordance with the Declaration of Helsinki and its later amendments. Because of the retrospective design of the study, the requirement for informed consent was waived by the ethics committee.

### 2.3. Data Source and Image Archive

The study material consisted of panoramic radiographs retrieved from the digital imaging archive of patients who had undergone CBCT examination for various clinical indications and who had a panoramic radiograph from the same period. On this basis, 500 panoramic radiographs with corresponding CBCT images from the same patients were retrospectively identified.

Images were included if the panoramic radiograph and the CBCT scan belonged to the same patient, both imaging modalities were of adequate diagnostic quality, and the images were suitable for evaluating the predefined incidental findings included in the study. Each panoramic radiograph was evaluated separately for each of the nine incidental findings. The presence or absence of each finding was confirmed using the corresponding CBCT images and recorded in a structured, finding-level database.

### 2.4. Imaging Equipment and Technical Parameters

Panoramic radiographs were acquired using a Veraviewepocs 3D R100/F40 unit (J. Morita Mfg. Corp., Kyoto, Japan). CBCT images were acquired using a NewTom 5G unit (QR s.r.l., Verona, Italy). Depending on the clinical indication and the anatomical region examined, fields of view (FOV) of 8 × 8, 12 × 8, 15 × 12, or 18 × 16 cm were used. Images were acquired at a tube voltage of 110 kVp, with the tube current set by the device’s automatic dose-control system, and with isotropic voxel sizes ranging from 0.20 to 0.30 mm depending on the protocol used. Because the imaging field was centered on the maxilla, mandible, or the relevant maxillofacial region according to the clinical indication for the examination, anatomical coverage could vary between patients. Before inclusion, each panoramic radiograph–CBCT pair was checked to confirm that the relevant anatomical area could be adequately assessed on the CBCT sections for each of the nine findings examined. Only cases for which a valid CBCT reference assessment could be made for all nine findings were included in the study, and no patient–finding unit was excluded because of inadequate coverage of the imaging field.

### 2.5. Sample Selection and Eligibility Criteria

The purpose of this study was not to determine the prevalence of incidental findings in a routine clinical population, but to compare the diagnostic performance of human and LLM readers in detecting nine predefined findings on panoramic radiographs. The study sample therefore consisted of a retrospectively assembled, finding-enriched diagnostic accuracy dataset designed to ensure adequate representation of the findings examined, rather than a consecutive clinical patient series.

Archival records obtained between January 2021 and December 2025 that contained both a panoramic radiograph and CBCT images from the same patient were screened for eligibility. Only cases in which the panoramic radiograph and the CBCT examination were performed on the same day were considered eligible. Both the panoramic radiograph and the CBCT images were required to be of adequate diagnostic quality, with the relevant anatomical areas suitable for evaluation for each of the nine findings examined. Cases with inadequate diagnostic quality, motion artifact, or positioning error, those lacking a corresponding CBCT image, or those in which the CBCT imaging field did not allow a valid reference assessment for all nine findings were excluded. Only one panoramic radiograph–CBCT pair per patient was included in the study.

Eligible cases were classified according to the nine findings examined on the basis of the CBCT-based reference assessment. Sample selection was performed by an oral and maxillofacial radiologist who was a member of the three-person CBCT reference assessment team but did not take part in the expert evaluation of the panoramic radiographs. Finding-based stratified sampling was applied to ensure adequate representation of the nine findings, and cases within each stratum were selected from the eligible case pool using a computer-generated random number sequence. The visibility, size, typicality, or diagnostic difficulty of the findings on the panoramic radiograph were not taken into account during selection. Sample selection was completed before the expert and LLM evaluations, and radiographs containing more than one finding were permitted.

**Figure 1:**
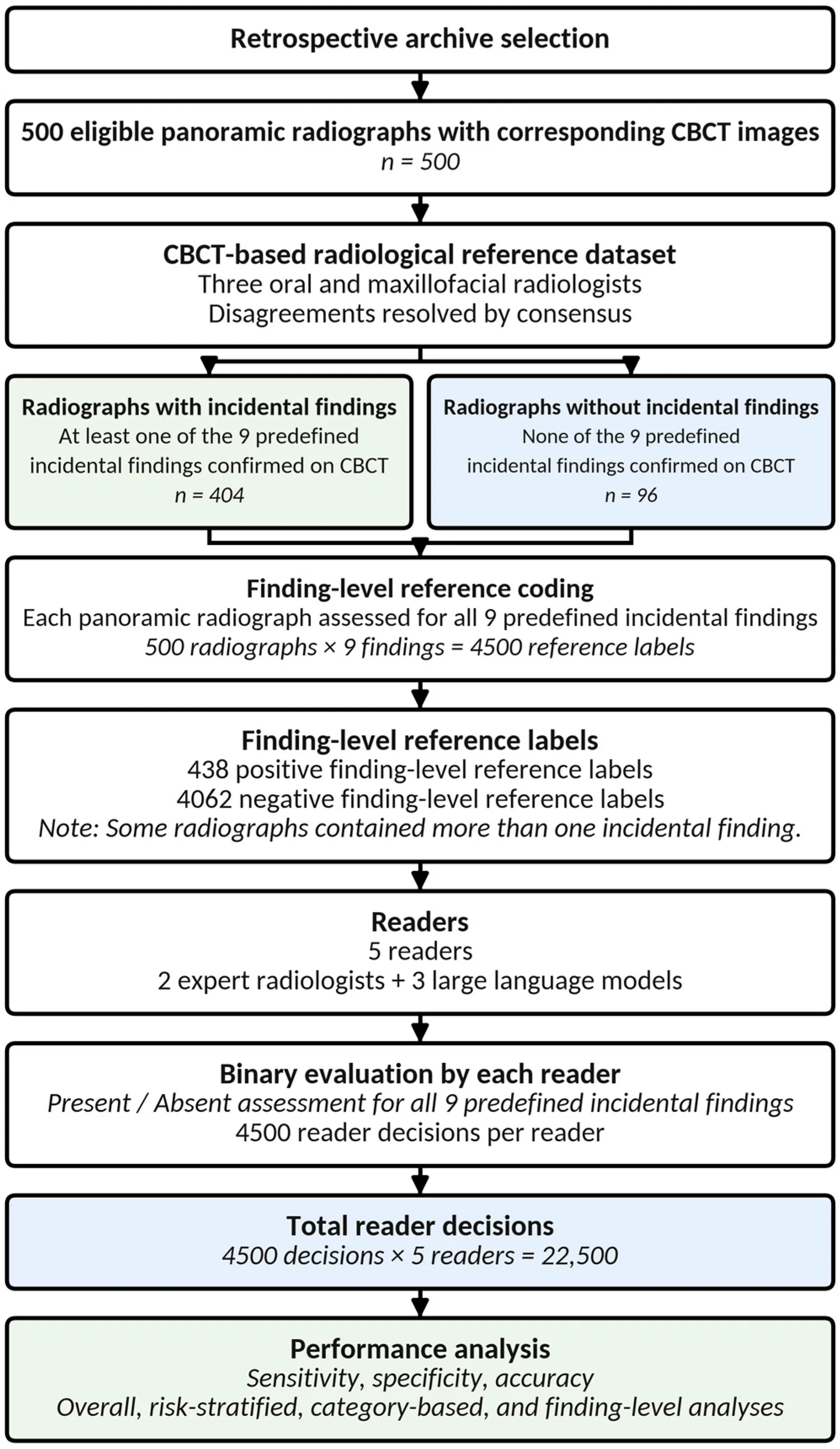
Simplified workflow of dataset preparation, the evaluation process, and performance analysis.

Radiographs in which none of the nine findings examined was present on the CBCT-based reference assessment were also randomly selected from the eligible negative-case pool and included in the dataset. The final enriched dataset comprised 500 panoramic radiographs. Of these, 404 contained at least one of the nine findings examined, while 96 contained none. Because each panoramic radiograph was evaluated separately for all nine findings, a total of 4500 finding-level binary decisions were obtained for each reader. According to the CBCT-based reference standard, 438 of these decisions were positive and 4062 were negative. The distribution of the 438 finding-level positive decisions was as follows: 46 for tonsillolith, 56 for mild mucous retention pseudocyst, 55 for enostosis, 50 for residual root, 54 for condensing osteitis, 45 for sialolith, 37 for carotid artery calcification, 55 for extensive maxillary sinus pathology, and 40 for large osseous lesion. The dataset construction and evaluation process are shown in Figure 1.

No formal a priori sample size or power calculation was performed. The sample size was determined pragmatically by the number of eligible panoramic radiograph–CBCT pairs available in the archive, by the aim of obtaining an adequate number of CBCT-confirmed positive cases for each of the nine findings, and by the feasibility of performing 1500 single-session LLM evaluations. All diagnostic performance estimates are therefore reported with 95% confidence intervals, and the finding-level comparisons should be interpreted with the relatively small number of positive cases per finding (37–56) in mind.

### 2.6. CBCT Reference Assessment

CBCT images were used as the reference imaging modality for the radiological confirmation of the nine incidental findings examined in the study and for the diagnostic performance analyses. The reference assessment was performed by three radiologists with 12, 9, and 7 years of experience in oral and maxillofacial radiology, respectively, none of whom took part in the evaluation of the panoramic radiographs. Radiological criteria for the presence and absence of each incidental finding were predefined before the assessment.

CBCT volumes were identified by anonymous study codes and randomly ordered. Reference assessors were blinded to the panoramic radiographs, to the expert and LLM evaluation results, and to clinical information. Each CBCT volume was systematically examined for all nine predefined incidental findings, irrespective of whether a suspicious area had been identified on the corresponding panoramic radiograph. Assessments were performed on axial, coronal, and sagittal sections using a structured evaluation form.

The three radiologists performed their initial assessments independently of one another. In cases of disagreement, only the relevant CBCT sections were re-examined jointly, and a final decision was reached by consensus. Panoramic radiographs and the expert or LLM evaluation results were not used during the consensus process. The final CBCT-based reference dataset was therefore constructed independently of the index test results.

### 2.7. Definition of Incidental Findings

In this study, incidental findings were defined as findings on panoramic radiographs that were unrelated to the patient’s presenting complaint or primary imaging indication but that could carry clinical or radiological significance.

The incidental findings included in the study were tonsillolith, mild mucous retention pseudocyst, enostosis, residual root, condensing osteitis, sialolith, carotid artery calcification, extensive maxillary sinus pathology, and large osseous lesion.

Before evaluation, the nine incidental findings examined in the study were operationally defined on the basis of anatomical location, radiographic morphology, relationship to adjacent teeth and anatomical structures, multiplanar appearance on CBCT, and, where applicable, size or extent criteria. The operational criteria used for the panoramic radiograph and CBCT assessments are presented in Table 1.

The operational definitions presented in Table 1 were developed to delineate the boundaries of the finding classes before evaluation and to standardize the CBCT-based reference assessment. No additional diagnostic criteria or example images were provided to the experts or LLMs evaluating the panoramic radiographs beyond the standardized prompt.

### 2.8. Risk-Based Classification

**Figure 2:**
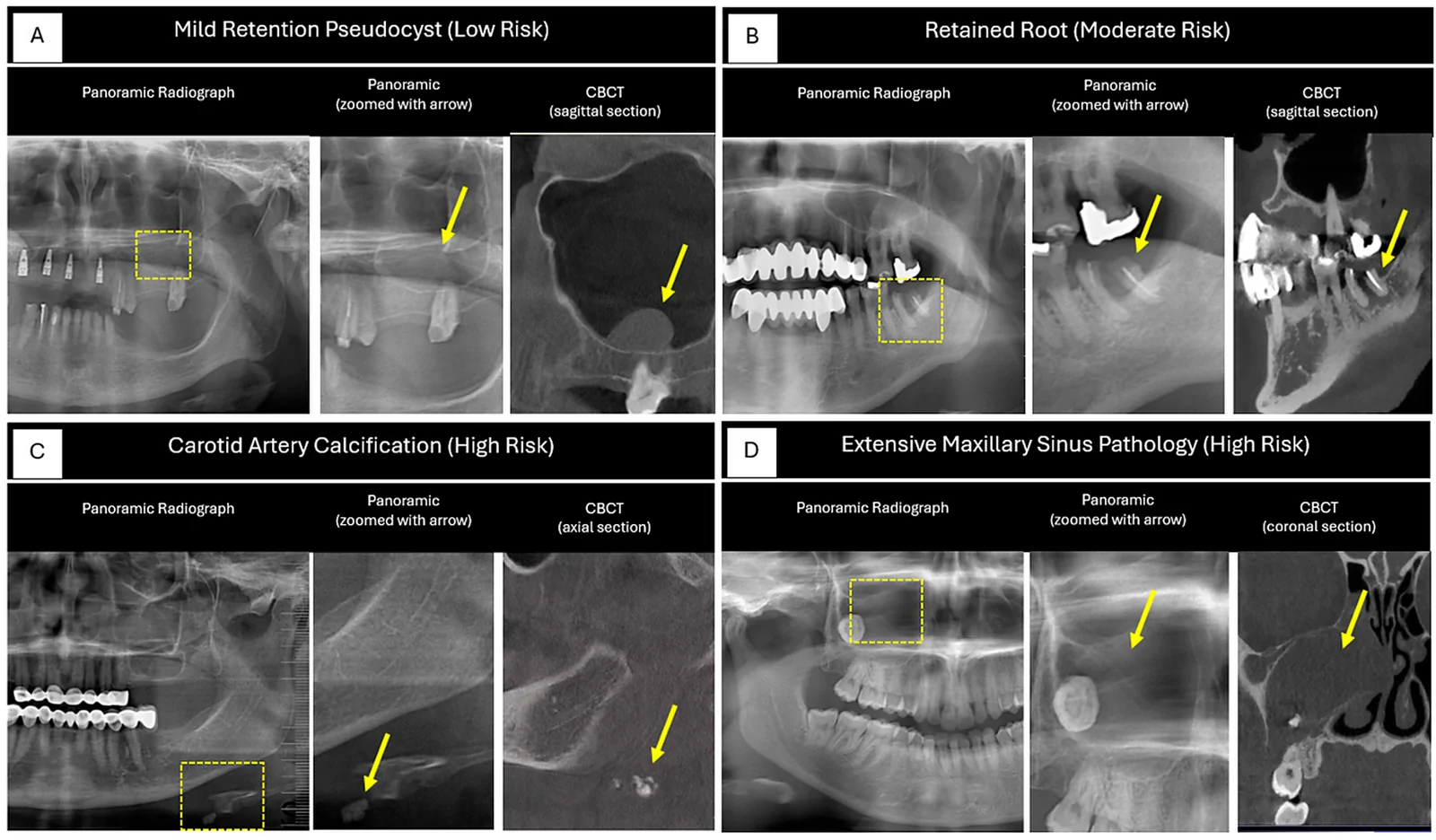
Representative CBCT-confirmed incidental findings included in the study. (**A**) mild retention pseudocyst, (**B**) residual root, (**C**) carotid artery calcification, and (**D**) extensive maxillary sinus pathology. For each lesion, the corresponding panoramic radiographic image, a magnified panoramic view, and the relevant CBCT sections are shown. Dashed lines and arrows indicate the lesion sites. CBCT: cone-beam computed tomography.

Because no universally accepted standard risk classification exists for incidental findings detected on panoramic radiographs, the risk groups used in this study were developed specifically for subgroup performance analyses rather than for the primary diagnostic analysis. This classification was not intended to determine the clinical prognosis of the findings or to propose a universal risk-grading system. Risk groups were determined by consensus among the three oral and maxillofacial radiology specialists on the CBCT reference assessment team, taking into account the findings’ probable clinical significance, need for follow-up, likelihood of requiring additional imaging, and need for referral to the relevant specialty. Because this grouping rests on expert consensus rather than on a validated classification system, all risk-level results were prespecified as exploratory subgroup analyses. Representative, CBCT-confirmed examples of incidental findings corresponding to the different risk groups are shown in Figure 2.

Low-risk findings were considered those that generally do not require urgent clinical intervention and can usually be managed with routine clinical evaluation or follow-up. Tonsillolith, mild mucous retention pseudocyst, and enostosis were included in this group [10,26,27,28,29]. Within this group, tonsilloliths are usually asymptomatic dystrophic calcifications that rarely require treatment; mild mucous retention pseudocysts are typically self-limiting and need no intervention in the absence of symptoms; and enostosis is a developmental variant of normal bone that requires neither treatment nor further imaging once recognized.

Moderate-risk findings were defined as those that may require clinical correlation or a relevant dental evaluation. Residual root, condensing osteitis, and sialolith were included in this group [10,27,28]. Within this group, residual roots may act as a focus of infection and usually require a decision on removal; condensing osteitis indicates a persistent inflammatory dental source that calls for endodontic or restorative assessment; and sialoliths may cause obstructive symptoms and generally warrant clinical evaluation of the affected gland.

High-risk findings were classified as clinically more significant findings that may require further evaluation, follow-up, or referral to the relevant specialty. Carotid artery calcification, extensive maxillary sinus pathology, and large osseous lesion were included in this group [10,27,28,30]. Within this group, carotid artery calcification is a possible marker of atherosclerotic disease and cerebrovascular risk that warrants medical referral; extensive maxillary sinus pathology may reflect sinusitis, polyposis, or, rarely, neoplasia and generally requires otorhinolaryngological or further radiological evaluation; and large osseous lesions require characterization, often with additional imaging and histopathological confirmation, because of their potential for local destruction or neoplastic behavior.

### 2.9. Anatomical and Clinical Category Classification

Incidental findings were further divided into four categories according to anatomical location and clinical characteristics [10,27,28,29]. Residual root and condensing osteitis were classified as dental findings; tonsillolith, sialolith, and carotid artery calcification as soft-tissue-related findings; mild mucous retention pseudocyst and extensive maxillary sinus pathology as maxillary sinus-related findings; and enostosis and large osseous lesion as osseous findings.

### 2.10. Large Language Models and Interfaces Used

Three commercial multimodal large language models capable of accepting image input were evaluated in this study: Claude Sonnet 4.6 (Anthropic, San Francisco, CA, USA), Gemini 3 (Google, Mountain View, CA, USA), and ChatGPT-5.3 (OpenAI, San Francisco, CA, USA). Evaluations were performed between 1 May and 1 June 2026, using the models’ consumer-facing, web-based chat interfaces. Claude Sonnet 4.6 was accessed via a Claude Pro subscription, Gemini 3 via a Google AI Pro subscription, and ChatGPT-5.3 via a ChatGPT Plus subscription. The model names displayed in the model-selection field of each platform, together with the access dates, were recorded during evaluation. In the Results and Discussion, these models are referred to as LLM 1 (Claude Sonnet 4.6), LLM 2 (Gemini 3), and LLM 3 (ChatGPT-5.3), respectively.

Models were not accessed through an application programming interface (API), and evaluations were performed under the platforms’ default operating settings. Because the consumer interfaces used did not allow the user to fix or verify parameters such as temperature, top-p, random seed, model snapshot, or image preprocessing, these parameters could not be controlled. No web browsing, plugins, external tools, project features, or model-specific additional operating modes were used during evaluation.

### 2.11. Preparation of Images Presented to the LLMs

Panoramic radiographs were exported from the digital imaging archive in JPEG format. Because the native export resolution was not consistently recorded in the retrospective archive, and to provide a standardized presentation across readers and platforms, all images were rescaled to 1536 × 832 pixels while preserving the original aspect ratio and saved with medium-level JPEG compression. A single, moderate pixel size was chosen because the consumer interfaces impose their own, undisclosed limits on the size and resolution of uploaded images and may resize them internally before they reach the model; presenting identical files to all three platforms was intended to ensure that the models received the same inputs rather than inputs that each platform had downscaled differently. The same rescaled files were also presented to the expert readers, so that human and model readers evaluated identical image content. No additional compression, sharpening, contrast adjustment, or artificial image enhancement was applied beyond this step. The rescaling nevertheless reduced pixel resolution relative to the original archive images, and the resulting loss of fine detail may have lowered the detectability of small or low-contrast findings for all readers; this is considered further in Section Limitations.

Patient names, identification numbers, and other identifying information were removed, and each image was assigned an anonymous, study-specific code. The diagnostic anatomical field of the image was preserved during anonymization, and no markings, arrows, or explanatory text were added to the images. The same anonymized JPEG files, unchanged in file format, pixel dimensions, and visual content, were uploaded separately to the consumer-facing web interface of each of the three LLMs.

Because the platforms did not provide technical information on any internal resizing or compression applied to uploaded images before processing by the model, the final resolution of the images actually processed by the models could not be verified.

### 2.12. Image Evaluation Protocol for the Large Language Models

Before evaluation, the 500 panoramic radiographs were randomly ordered by computer according to their anonymous study codes. To standardize image presentation across models, the same randomized image order was used for all three models. Each panoramic radiograph was evaluated in a new, independent chat session for each model. Accordingly, 500 independent chat sessions were generated for each model, for a total of 1500 across the three models. Only one panoramic radiograph was evaluated per chat session.

Chat history, personalization, and memory features were disabled during evaluation to the extent permitted by each platform. Previous images, prompts, or model responses were not present in the conversation context of any new session. The same standardized prompt was presented to the models, unchanged, across all evaluations. No additional prompt, explanation, hint, feedback, or correction was provided after the model’s initial response, and the model was not asked to re-evaluate its answer.

The models were not given information on patient age, sex, clinical history, imaging indication, presumptive diagnosis, reference findings, or CBCT results. Each image was evaluated only once, under a predefined, single-pass protocol. The models’ first, unassisted responses were therefore included in the analysis.

Because evaluations were performed through continuously updatable consumer interfaces rather than an API, the results represent the performance of the model versions available during the stated access period, under the defined interface, image presentation, and prompt protocol, rather than the general and fixed diagnostic capacity of the respective models; because these interfaces and the underlying models are updated over time, performance may differ in later evaluations.

### 2.13. Prompt Standardization

The same instructional prompt was used for all models, and the prompt content was not altered during evaluation. Models were asked to provide only a structured “Present/Absent” response for the nine incidental findings included in the study; free-text commentary, diagnostic suggestions, or clinical recommendations were not requested. The standardized prompt was as follows:

*“Please evaluate the presented panoramic radiograph only for the incidental findings listed below. For each finding, indicate only one of the following two options: ‘Present’ or ‘Absent’. Please do not provide any additional explanation, commentary, diagnostic suggestion, or clinical advice. Findings to be evaluated: Tonsillolith | Mild mucous retention pseudocyst | Enostosis | Residual root | Condensing osteitis | Sialolith | Carotid artery calcification | Extensive maxillary sinus pathology | Large osseous lesion.”*

### 2.14. Coding of Model Responses

Model responses were converted to binary format according to a predefined coding scheme. Responses indicating the presence of a finding were coded as “1” and those indicating its absence as “0”. Only structured responses were evaluated, and no post hoc interpretation or reclassification was performed. All model responses were unambiguous and codable for all nine findings; consequently, there were no invalid responses, missing data, reclassifications, or model decisions excluded from analysis.

### 2.15. Expert Evaluation Protocol

Two oral and maxillofacial radiologists with 5 and 6 years of experience, respectively, took part in the evaluation process. Both were specialists in oral and maxillofacial radiology who had completed the national three-year specialty training program in this field, one holding a faculty position and the other a clinical specialist position at the same institution. The stated years of experience refer to their total experience in oral and maxillofacial radiology, including the specialty training period, and both readers interpret panoramic radiographs and CBCT examinations as part of their routine clinical work. The term “expert” is used throughout the manuscript to denote these qualified specialist readers, as distinct from the LLM readers. The experts assessed all panoramic radiographs in a blinded manner and independently of each other. The expert readers were presented with the same anonymized, 1536 × 832-pixel JPEG files that had been uploaded to the LLM interfaces. Images were anonymized, randomly ordered, and presented in a digital environment. Assessments were performed under standardized viewing conditions, using the same monitor and the same imaging software.

Basic image adjustments such as brightness, contrast, and magnification were permitted, but advanced image-processing techniques were not used, and no time restriction was imposed on image interpretation.

Expert readers were not given information on patient age, sex, clinical history, imaging indication, presumptive diagnosis, CBCT findings, or the large language models’ responses. Assessments were based solely on the panoramic radiographs.

Although the same panoramic radiographic content was presented to all readers to enhance comparability, the reading conditions were not identical: the experts could adjust brightness, contrast, and magnification and had no time limit, whereas the LLMs received the images through consumer interfaces with undisclosed internal image processing. The implications of this asymmetry are addressed in Section Limitations.

### 2.16. Coding of Expert Responses

Experts were asked to evaluate each of the nine predefined incidental findings separately as “present” or “absent” for every panoramic radiograph; the same standardized prompt used for the LLMs was also used for the expert evaluation. Expert responses were recorded directly as “Present” or “Absent” using a structured form. Responses indicating the presence of the corresponding finding were coded as “1” and those indicating its absence as “0”. Because responses were collected in binary format, no post hoc interpretation or reclassification was performed.

### 2.17. Interobserver Agreement

Interobserver agreement was evaluated on the basis of the 4500 finding-level “present/absent” decisions made by the two expert radiologists for the nine findings across the 500 panoramic radiographs. Cohen’s kappa coefficient was calculated for this purpose. Kappa values were interpreted as follows: <0.20, poor agreement; 0.21–0.40, fair agreement; 0.41–0.60, moderate agreement; 0.61–0.80, good agreement; and 0.81–1.00, very good agreement.

### 2.18. Statistical Analysis

Categorical data were summarized as counts and percentages. Interobserver agreement between the two experts’ finding-level binary decisions was assessed using the unweighted Cohen’s kappa coefficient. Agreement among the three reference radiologists’ independent, pre-consensus decisions was examined using Fleiss’ kappa coefficient. In addition, the number and percentage of patient–finding units in which at least one reference assessor’s decision differed from the others were calculated. With the CBCT assessment taken as the reference standard, sensitivity, specificity, accuracy, false-negative rate, and false-positive rate were calculated for each reader. Diagnostic performance metrics were presented as point estimates with 95% confidence intervals.

The evaluations of the nine findings within the same patient were assumed not to be independent of one another, owing to shared anatomical and imaging characteristics. The 95% confidence intervals for the diagnostic performance metrics and for Cohen’s and Fleiss’ kappa coefficients were therefore calculated using a patient-level, nonparametric cluster bootstrap. The patient identifier was used as the resampling unit; the nine findings from the same patient, all reader evaluations, and the reference assessors’ independent decisions were kept together within each bootstrap sample. A total of 5000 resamples were performed, and 95% confidence intervals were determined using the percentile method. The bootstrap was used exclusively to estimate confidence intervals; resampling does not increase the number of observations available for inference, because each bootstrap sample has the same size as the original dataset (500 patients) and the resampling distribution serves only to quantify the sampling uncertainty of the point estimates while preserving the within-patient correlation structure. All hypothesis tests (GEE models, Cochran’s Q tests, and exact McNemar tests) were performed on the original, unresampled data.

The readers’ overall diagnostic performance was compared using generalized estimating equations (GEE) with a binomial distribution and a logit link function. In the GEE models, the dependent variable was coded as “1” for correct classification and “0” for incorrect classification. The patient identifier was defined as the clustering variable, and an exchangeable working correlation structure was used. Reader and finding type were included in the models as fixed effects. Sensitivity analyses were performed separately using only observations positive on the reference standard, specificity analyses using only negative observations, and accuracy analyses using all observations. Cluster-robust sandwich standard errors were used for the parameter estimates. The overall reader effect was assessed using an omnibus Wald chi-square test for the reader fixed effect. In models where the overall reader effect was significant, pairwise comparisons were performed for all 10 reader pairs, including the three comparisons among the LLMs, and expressed as odds ratios (ORs) with 95% confidence intervals; Bonferroni correction was applied to these 10 comparisons.

Sensitivity analyses by risk level and finding category were performed using separate models within the same GEE framework. In these models as well, the patient identifier was used as the clustering variable, and the overall reader effect was assessed using an omnibus Wald chi-square test. The 95% confidence intervals for the risk- and category-level sensitivity estimates were calculated using the patient-level cluster bootstrap. These analyses were considered secondary and exploratory; no additional correction for multiple testing was applied to the omnibus *p*-values, and the results were interpreted with caution.

For the finding-specific sensitivity analyses, the paired results of the five readers on the same cases were compared using Cochran’s Q test. To control for the multiple-testing problem arising from the nine Cochran’s Q tests performed across findings, *p*-values were corrected using the Benjamini–Hochberg false discovery rate procedure. For findings that remained significant after correction, pairwise comparisons of all 10 reader pairs were performed using the exact McNemar test, with Bonferroni correction applied to the within-finding comparisons. The 95% confidence intervals for the finding-specific sensitivity values were obtained using the patient-level cluster bootstrap.

No inferential test treating the 4500 patient–finding observations across the nine findings as independent data points was applied. Patient-level clustering was accounted for in the inferential comparisons through the GEE models. Descriptive statistics, the point estimates of Cohen’s and Fleiss’ kappa coefficients, the GEE models, Cochran’s Q and exact McNemar tests, and the multiple-comparison corrections were performed using IBM SPSS Statistics for Windows, version 26.0 (IBM Corp., Armonk, NY, USA). The patient-level cluster bootstrap procedure was carried out using Python software (version 3.12.13; Python Software Foundation, Wilmington, DE, USA). The random-number generator seed was fixed at 2026 to ensure the reproducibility of the bootstrap analyses. All tests were two-tailed. Statistical significance was set at *p* < 0.05, and at q < 0.05 for analyses to which the Benjamini–Hochberg correction was applied.

## 3. Results

A total of 500 panoramic radiographs were examined separately by each reader for each of the nine predefined incidental findings. Accordingly, 4500 finding-level binary decisions were analyzed for each reader, yielding a total of 22,500 finding-level binary decisions across the five readers.

### 3.1. Interobserver Agreement

Interobserver agreement between the two expert readers was assessed on the basis of the finding-level “present/absent” decisions for the nine predefined incidental findings on the panoramic radiographs. The unweighted Cohen’s kappa coefficient between Expert 1 and Expert 2 was κ = 0.86 (95% CI: 0.84–0.88), indicating very good interobserver agreement.

A high level of agreement was found among the three reference radiologists’ independent, pre-consensus, finding-level assessments (Fleiss’ κ = 0.89; 95% CI: 0.87–0.91). In 144 of the 4500 patient–finding units (3.2%), at least one reference assessor’s decision differed from the others. These units were resolved through joint review and consensus.

### 3.2. Overall Diagnostic Performance

Evaluation of the nine target findings across the 500 panoramic radiographs yielded 4500 patient–finding pairs. According to the CBCT reference standard, 438 of these observations were positive and 4062 were negative. The readers’ overall diagnostic performance is shown in Table 2. The highest sensitivity, specificity, and accuracy were observed for Expert 1, while the lowest sensitivity and accuracy were observed for LLM 3. False-negative rates were lower for the experts than for the LLMs.

In the GEE analysis accounting for within-patient clustering, the reader effect was significant for sensitivity [Wald χ^2^(4) = 51.81; *p* < 0.001], specificity [Wald χ^2^(4) = 87.51; *p* < 0.001], and accuracy [Wald χ^2^(4) = 127.40; *p* < 0.001]. In Bonferroni-corrected pairwise comparisons, Expert 1’s sensitivity was significantly higher than that of all three LLMs. Expert 2 showed higher sensitivity than LLM 2 and LLM 3, whereas the difference between Expert 2 and LLM 1 did not remain significant after correction. Both experts’ specificity and accuracy values were higher than those of all three LLMs. No significant difference was found between the two experts for any of the performance metrics evaluated. Among the three LLMs, LLM 1 showed significantly higher sensitivity than LLM 3 (OR 1.65, 95% CI 1.17–2.32; Bonferroni-adjusted *p* = 0.039), and LLM 2 showed significantly higher accuracy than LLM 3 (OR 1.23, 95% CI 1.08–1.40; adjusted *p* = 0.022); none of the other pairwise comparisons among the LLMs remained significant after correction (Table 2; full pairwise results are given in Supplementary Table S1).

### 3.3. Sensitivity by Risk Level

In the exploratory risk-level analyses, sensitivity for low-risk findings was numerically higher for the experts, and the overall reader effect was significant (*p* = 0.010). No significant difference between readers was found in the moderate-risk group (*p* = 0.166). The most pronounced difference was observed for high-risk findings, for which the overall reader effect was significant (*p* < 0.001). In this group, sensitivity was numerically higher for the experts than for the LLMs. Among the LLMs, the highest sensitivity was observed for LLM 1 and the lowest for LLM 3 (Table 3).

**Figure 3:**
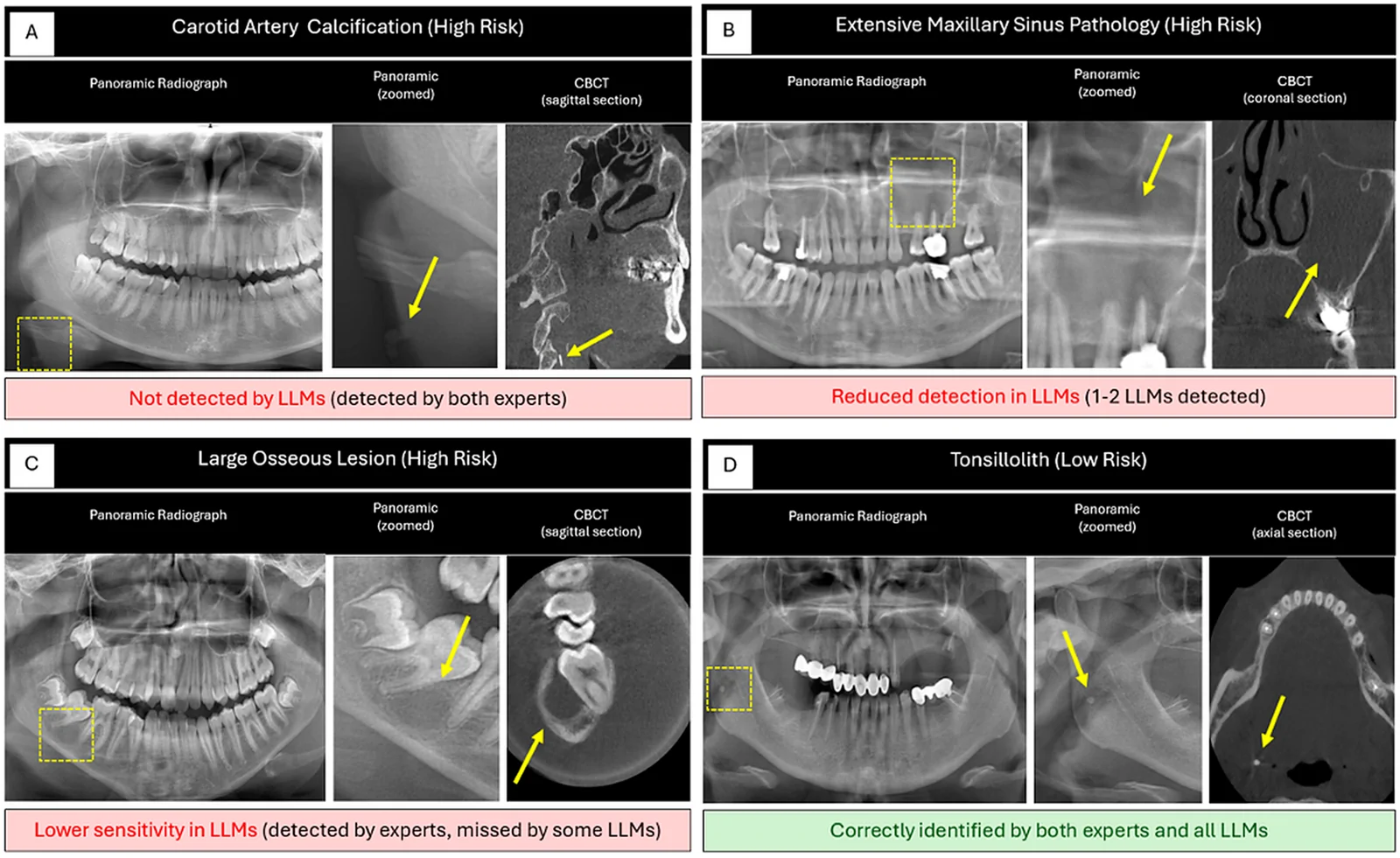
Representative cases illustrating diagnostic performance differences between expert readers and large language models (LLMs). (**A**) Carotid artery calcification detected by both experts but missed by the LLMs, (**B**) extensive maxillary sinus pathology with a low detection rate among the LLMs, (**C**) a large osseous lesion with lower sensitivity on LLM assessment, and (**D**) a tonsillolith case correctly identified by both the experts and all LLMs. For each case, the panoramic radiograph, a magnified image, and the corresponding CBCT sections are shown. Dashed boxes outline the regions presented in the magnified images. Arrows indicate the sites of the respective findings. CBCT: cone-beam computed tomography.

Representative cases illustrating the diagnostic performance differences between the experts and the LLMs are presented in Figure 3.

### 3.4. Sensitivity by Finding Category

Sensitivity values by finding category are shown in Table 4. The difference between readers was significant for soft-tissue and sinus findings (*p* < 0.001 for both). In both categories, the highest sensitivity was achieved by the experts. In contrast, the reader effect did not reach statistical significance for osseous findings (*p* = 0.070) or dental findings (*p* = 0.204).

### 3.5. Finding-Level Sensitivity

After Benjamini–Hochberg correction was applied to the tests performed for the nine findings, the difference in sensitivity between readers remained significant only for carotid artery calcification (q < 0.001) and extensive maxillary sinus pathology (q = 0.005). For both findings, expert sensitivity was numerically higher than that of the LLMs. The most pronounced performance difference between readers was observed for carotid artery calcification. In Bonferroni-adjusted pairwise exact McNemar comparisons for this finding, both experts were more sensitive than LLM 2 (Expert 1, adjusted *p* = 0.013; Expert 2, adjusted *p* = 0.023) and LLM 3 (Expert 1, adjusted *p* < 0.001; Expert 2, adjusted *p* = 0.004), whereas the comparisons between the experts and LLM 1 and all comparisons among the three LLMs were not significant. For extensive maxillary sinus pathology, only the comparisons of Expert 1 (adjusted *p* = 0.015) and Expert 2 (adjusted *p* = 0.026) with LLM 3 remained significant, and the LLMs did not differ significantly from one another (Table 5).

For tonsillolith, mild mucous retention pseudocyst, enostosis, residual root, condensing osteitis, sialolith, and large osseous lesion, the differences between readers were not significant after correction for multiple comparisons. The number of positive observations per finding ranged from 37 to 56 (Table 5).

## 4. Discussion

In this finding-enriched dataset, the two expert readers achieved higher overall diagnostic performance than the three multimodal LLMs in detecting nine incidental findings on panoramic radiographs. The gap was widest in the exploratory high-risk group; finding-level analyses trace most of it to carotid artery calcification and extensive maxillary sinus pathology. For the moderate-risk findings, and for seven of the nine findings overall, the differences between readers did not reach statistical significance after correction for multiple comparisons. Differences among the three LLMs themselves were small (Section 3.2).

The lower performance of the LLMs is consistent with the known limitations of general-purpose LLMs in radiological image interpretation, where visual interpretation, hallucinated content, and the need to verify outputs remain recurring concerns [31]. The gap between text-based competence and image interpretation is well documented: ChatGPT performs reasonably on radiology board-style questions but poorly when imaging findings must be described [20,21], handles overt pathology better than subtle findings [32], and shows low accuracy for third-molar assessment on panoramic radiographs [33]; Liu et al. likewise reported high sensitivity for overt radiopaque features but markedly lower sensitivity for caries and periapical lesions [22], and a systematic review concludes that image-based tasks in dentomaxillofacial radiology still require expert oversight [23]. Even task-specific deep learning systems perform unevenly across panoramic findings [25,34], and their accuracy depends on the imaging modality, target finding, dataset, and model architecture [13,14,15]; general-purpose LLMs, which were not trained for dental radiology, should therefore not be expected to match purpose-built models.

Because only 438 of the 4500 patient–finding pairs were positive, accuracy and specificity are inevitably weighted toward the negative observations, and, as Liu et al. also warned, accuracy alone can mislead in datasets dominated by the negative class [22]. Reporting sensitivity and false-negative rates separately gives a more balanced picture: the false-negative rates were 8.4–10.5% for the experts against 16.9–24.9% for the LLMs, a gap that matters most where the missed finding is clinically important.

The exploratory risk-level analysis brings the clinical stakes of this difference into sharper focus, with the caveat that the risk groups were defined by consensus for this study and do not represent an established clinical risk classification. Readers did not differ significantly in the moderate-risk group, whereas expert sensitivity for the findings grouped as high-risk stayed above 90%, against 62.9–78.0% for the LLMs: an LLM alone could miss such a finding in roughly one case in four to one in three. Because these findings were deliberately over-sampled, this is a miss rate per positive case, not the number of missed findings to be expected in a routine clinical population, in which they are considerably less common. Carotid artery calcification and extensive maxillary sinus pathology account for most of this gap; large osseous lesions showed a similar trend that did not survive FDR correction, so the group-level difference should not be read as applying equally to every finding within it.

Carotid artery calcification showed the largest sensitivity gap in our study. Its prevalence and clinical significance on panoramic radiographs are well documented [6], along with associations with age, missing teeth, hypertension, and other vascular risk factors [7,35]. The LLMs’ low sensitivity here may simply be visual: these calcifications appear as small, irregular radiopacities, and distinguishing them from other cervical soft-tissue calcifications depends on recognizing their anatomical location rather than any single defining feature. Reading conditions may also have contributed: the experts could magnify the image and adjust its contrast, whereas the LLMs received the same files through interfaces with undisclosed internal processing, so part of the gap for such small, faint calcifications may reflect the conditions of comparison rather than model capability alone.

A similar explanation may apply to extensive maxillary sinus pathology. Superimposition of the zygomatic structures, maxillary teeth, and surrounding anatomy can make the sinus harder to assess in the first place [8,9], and CBCT studies have shown how common sinonasal anatomical variation and mucosal change are [36]. Distinguishing extensive opacification from physiological or limited mucosal change requires weighing its distribution and anatomical context rather than simply spotting an opacity, which may be exactly where the LLMs fell short relative to the experts in the sinus category. The same asymmetry in reading conditions applies to such subtle, low-contrast findings and should be kept in mind when this gap is interpreted.

That readers did not differ significantly after FDR correction for the remaining seven findings does not mean the LLMs matched the experts on them. With only 37–56 positive samples per finding, no formal a priori power calculation, and the higher statistical bar imposed by correction for multiple comparisons, some real differences may have gone undetected, especially since most of these findings still showed a numerically declining sensitivity trend from experts to LLMs; these null results are a failure to detect a difference in this dataset, not evidence of comparable performance.

LLMs’ fluency in generating text should also be separated from competence at interpreting images: dental radiology reports written with ChatGPT read well but have been shown to omit critical diagnostic information [24]. Taken together, our findings argue against using these models independently to evaluate panoramic radiographs under the conditions tested, because their false-negative rates for the findings grouped as high-risk were too high. Any place these systems may have in panoramic radiograph evaluation is more likely to be as an adjunct to the radiologist, for example as a preliminary screening or checklist aid that flags possible findings for expert confirmation, than as a replacement for expert interpretation. This study did not test such a combined workflow, so any adjunctive role would have to be established prospectively in clinically representative case series; because performance also varied across the three models and consumer-interface models change over time, each model version would need to be evaluated on its own terms.

The comparative design of this study, with 500 panoramic radiographs, a CBCT reference standard, two experts, and three LLMs, is one of its main strengths. CBCT allowed the uncertainty inherent in two-dimensional panoramic imaging to be resolved more reliably, and detection of CBCT-confirmed incidental findings on panoramic radiographs is known to vary by anatomical region, with sinus findings achieving only limited-to-moderate accuracy [37]; the high frequency of clinically relevant incidental findings in CBCT studies [2,3,4,5,10,11,12,26,27,28,29,30] also underlines that panoramic radiographs deserve evaluation beyond the teeth and alveolar structures [1,38]. The GEE and cluster bootstrap methods, which account for within-patient clustering, and the finding-level FDR correction add further weight to the statistical inferences.

### Limitations

This study has several limitations. First, the retrospective, single-center design, with all radiographs acquired on a single panoramic unit, limits how far the findings can be generalized to other patient populations, imaging systems, and clinical settings. Image characteristics differ between panoramic devices and acquisition protocols, and how consumer-interface LLMs respond to such differences is unknown, so the results require validation in multicenter, multi-device datasets. The sample was also an enriched dataset rather than a consecutive clinical series, built to ensure adequate representation of rare findings, which may have introduced spectrum bias; this is unlikely to change the relative ranking of readers but does bear on the size of the pooled difference, because the over-sampled high-risk findings showed the largest gap between readers, so the overall estimates cannot be transferred directly to routine clinical populations, in which predictive values would differ. The modest number of positive samples per finding (37–56) and the absence of a formal a priori sample size calculation may also have kept some genuine differences from reaching significance after correction for multiple testing.

Second, assessments relied on the panoramic radiographs alone, with no clinical information or imaging indication provided, and the comparison between the human and LLM readers was not fully balanced: the experts could adjust brightness, contrast, and magnification and had unlimited reading time, whereas the LLMs received the same files through consumer interfaces whose internal processing, resizing, and compression are undisclosed and could not be verified. This asymmetry is a major source of unevenness in the comparison and is most likely to have affected small calcifications and other subtle, low-contrast findings, for which magnification and contrast adjustment matter most; its direction and magnitude cannot be established from the present data. The rescaling of all images to 1536 × 832 pixels, applied to ensure identical inputs, likewise reduced pixel resolution relative to the archive images and may have lowered the detectability of such findings for all readers.

Third, the human comparator consisted of two specialist readers from a single institution. Although their interobserver agreement was very good (κ = 0.86), two readers cannot represent the range of performance among the clinicians who interpret panoramic radiographs, and their individual skill inevitably shaped the size of the expert–LLM difference; the results are therefore a comparison against two specific readers rather than against human expertise in general, and larger, more heterogeneous reader panels are needed before broader conclusions are drawn.

Fourth, CBCT is a radiological reference standard rather than a definitive one equivalent to independent clinical, surgical, or histopathological confirmation, and it may not establish the nature of every finding examined, particularly some osseous lesions and soft-tissue calcifications. The risk-based classification has a similar caveat: it rests on radiologist consensus rather than a validated classification system, so the risk-group results are best treated as exploratory rather than confirmatory.

Fifth, each model saw each radiograph only once, in a single independent session, so within-model repeatability could not be examined. Generative models produce their output stochastically, and the consumer interfaces did not allow the temperature, random seed, or model snapshot to be fixed, so the same image submitted again could receive a different answer. The reported values therefore carry an unquantified test–retest uncertainty beyond the sampling uncertainty expressed by the confidence intervals and should be read as single-run estimates; future studies should include repeated independent submissions of the same images, at least for a subset of the dataset, so that within-model agreement can be quantified and reported alongside diagnostic accuracy. For the same reasons, and as noted in Section 2.12, the results describe performance through the specific consumer interfaces and access period tested (May–June 2026) rather than a fixed diagnostic capacity.

Finally, the mandatory binary “present/absent” format left the models no way to express diagnostic uncertainty or to flag an image they could not assess, and alternative prompt designs were not tested. A forced binary choice may have penalized the LLMs disproportionately on faint or equivocal findings; because the same format was applied to the experts, the comparison itself remained symmetric, but a response option allowing “uncertain” or “not assessable” would reflect clinical decision-making more closely and could be explored in future work. These are, moreover, general-purpose LLMs rather than systems built for dental radiology, and task-specific AI could perform differently.

## 5. Conclusions

In this finding-enriched, single-center dataset, two expert readers achieved higher overall diagnostic performance than three multimodal large language models in detecting incidental findings on panoramic radiographs (sensitivity of 89.5–91.6% versus 75.1–83.1%, respectively). The gap was widest in the exploratory high-risk group (expert sensitivity of 90.9–93.2% versus LLM sensitivity of 62.9–78.0%), and at the level of individual findings it remained significant after correction for multiple comparisons only for carotid artery calcification and extensive maxillary sinus pathology. These results describe the models’ performance through the specific consumer interfaces and during the access period tested, not their fixed diagnostic capacity, and the absolute estimates cannot be transferred directly to routine clinical populations, in which these findings are less prevalent. Under the conditions tested, current general-purpose LLMs should not be used independently to evaluate panoramic radiographs for incidental findings. If these models have a future role in this task, it is more likely to be as an expert-supervised adjunct or screening aid than as a replacement for expert interpretation; such a combined workflow was not examined in this study and would need to be established prospectively, in clinically representative datasets and with repeated model evaluations.

## Figures and Tables

**Table 1:** Operational definitions of the incidental findings evaluated in the study.

Finding	Panoramic Radiography Criterion	CBCT Reference Criterion	Size/Extent Criterion
Tonsillolith	One or more well-defined radiopaque foci superimposed on the mid-region of the mandibular ramus and the oropharyngeal airway	A calcified focus identified in the palatine tonsil region, between the medial surface of the mandibular ramus and the lateral wall of the oropharyngeal airway, confirmed on multiplanar sections	No minimum size defined
Mild mucous retention pseudocyst	A localized, smoothly marginated, homogeneous, dome-shaped opacity arising from the floor or wall of the maxillary sinus, without a corticated border	A dome-shaped soft-tissue density arising from the floor or wall of the maxillary sinus, surrounded by an aerated sinus cavity, without bone destruction	Occupying less than one-third of the sinus cavity
Enostosis	A localized, homogeneous, well- to moderately well-defined radiopaque area within the jawbone, without a surrounding radiolucent halo, expansion, or an evident inflammatory dental focus	A hyperdense bone focus continuous with the trabecular bone, without expansion or cortical destruction, and without an inflammatory cause in the adjacent tooth	No minimum size defined
Residual root	A radiopaque structure with root morphology, located in an edentulous or partially edentulous alveolar region, without connection to a functional crown	A dental tissue fragment of dentin density within or upon the alveolar bone, showing root morphology and no connection to a crown	Presence of a root fragment, irrespective of size
Condensing osteitis	A localized radiopaque area associated with the apex of a tooth showing deep caries, an extensive restoration, endodontic treatment, or evidence of pulpal/periapical inflammation	Periapical bone sclerosis directly related to the apical region of the corresponding tooth, without expansion, together with a probable inflammatory dental source	No minimum size defined
Sialolith	One or more well-defined radiopaque foci along the expected anatomical course of a major salivary gland or duct	A calcified structure at the expected anatomical location of a salivary gland or duct, not continuous with bone, and distinguishable from other soft-tissue calcifications by its three-dimensional position	No minimum size defined
Carotid artery calcification	An irregular, nodular, linear, or heterogeneous radiopaque formation seen posteroinferior to the mandibular angle, typically at the level of the C3–C4 vertebrae	A calcified focus localized to the carotid space or the expected anatomical region of the carotid bifurcation, distinguishable from adjacent bone and other cervical calcifications	No minimum size defined
Extensive maxillary sinus pathology	Diffuse mucosal opacity affecting a substantial portion of the maxillary sinus, partial or total opacification, an air–fluid level, or extensive polypoid soft-tissue appearance	Diffuse mucosal thickening or soft tissue affecting more than one sinus wall; partial-to-total opacification or an air–fluid level	Involvement of at least one-third of the sinus cavity, involvement of more than one wall, or partial/total opacification
Large osseous lesion	An intraosseous lesion of radiolucent, radiopaque, or mixed density in the maxilla or mandible, distinguishable from normal anatomical structures, enostosis, and condensing osteitis. The lesion’s extent within the bone and any changes it produced in adjacent structures were taken into account.	The intraosseous location and maximum diameter of the lesion were confirmed on axial, coronal, and sagittal sections. Features such as cortical expansion, thinning, or perforation; tooth displacement or root resorption; and extension into adjacent anatomical structures were evaluated.	The minimum diameter for inclusion in the study was ≥5 mm. The classification as “large” was based on the joint assessment of the lesion’s diameter, its extent within the bone, and its effects on surrounding structures.

CBCT: cone-beam computed tomography. Operational definitions were developed by the authors for this study.

**Table 2:** Overall diagnostic performance of the readers, with cluster-robust 95% confidence intervals.

Metric	*n*	Expert 1	Expert 2	LLM 1	LLM 2	LLM 3	*p*
Sensitivity	438	91.6 ^a^ (88.8–94.1)	89.5 ^ab^ (86.5–92.3)	83.1 ^bc^ (79.5–86.5)	79.2 ^cd^ (75.4–82.9)	75.1 ^d^ (71.2–79.0)	<0.001
Specificity	4062	93.0 ^a^ (92.2–93.8)	92.0 ^a^ (91.2–92.8)	89.0 ^b^ (88.1–89.9)	90.0 ^b^ (89.1–90.9)	88.0 ^b^ (86.9–89.0)	<0.001
Accuracy	4500	92.9 ^a^ (92.1–93.6)	91.8 ^a^ (91.0–92.5)	88.4 ^bc^ (87.5–89.3)	89.0 ^b^ (88.0–89.9)	86.8 ^c^ (85.7–87.8)	<0.001
False-negative rate (FNR)	438	8.4 (5.9–11.2)	10.5 (7.7–13.5)	16.9 (13.5–20.5)	20.8 (17.1–24.6)	24.9 (21.0–28.8)	—
False-positive rate (FPR)	4062	7.0 (6.2–7.8)	8.0 (7.2–8.8)	11.0 (10.1–11.9)	10.0 (9.1–10.9)	12.0 (11.0–13.1)	—

Values are given as point estimates (95% CI) in percent. Confidence intervals were calculated using a patient-level, nonparametric cluster bootstrap with 5000 resamples. *p*-values were obtained from omnibus Wald chi-square tests for the overall reader effect in GEE models accounting for patient-level clustering. Because the FNR and FPR are the complements of sensitivity and specificity, respectively, they were not tested separately. Values within the same row that do not share a superscript letter differ significantly in Bonferroni-adjusted pairwise GEE comparisons (adjustment for 10 reader pairs). LLM 1–3 are defined in Section 2.10.

**Table 3:** Sensitivity by risk level, with cluster-robust 95% confidence intervals.

Risk Level	Positive Pairs (*n*)	Expert 1	Expert 2	LLM 1	LLM 2	LLM 3	*p*
Low	157	94.3 (90.4–97.6)	92.4 (88.0–96.3)	89.2 (84.3–93.7)	86.0 (80.3–91.2)	82.8 (76.7–88.4)	0.010
Moderate	149	87.2 (81.7–92.3)	85.2 (79.3–90.9)	81.2 (74.6–87.5)	79.9 (72.9–86.1)	77.9 (71.3–84.4)	0.166
High	132	93.2 (88.6–97.1)	90.9 (85.8–95.6)	78.0 (70.6–84.9)	70.5 (62.7–78.1)	62.9 (54.9–70.8)	<0.001

Values are given as sensitivity (95% CI). Confidence intervals were calculated using the patient-level cluster bootstrap. *p*-values were obtained from omnibus Wald chi-square tests for the overall reader effect in GEE models accounting for patient-level clustering. LLM 1–3 are defined in Section 2.10.

**Table 4:** Sensitivity by finding category, with cluster-robust 95% confidence intervals.

Category	Positive Pairs (*n*)	Expert 1	Expert 2	LLM 1	LLM 2	LLM 3	*p*
Soft tissue	128	90.6 (85.2–95.5)	88.3 (82.4–93.6)	80.5 (73.1–87.1)	75.0 (67.4–82.1)	71.1 (63.1–78.9)	<0.001
Sinus	111	93.7 (88.9–97.7)	91.9 (86.2–96.5)	83.8 (76.7–90.3)	79.3 (71.6–86.7)	73.9 (65.5–82.0)	<0.001
Osseous	95	93.7 (88.5–98.0)	91.6 (85.6–96.7)	87.4 (80.2–93.6)	84.2 (76.2–91.5)	78.9 (69.8–87.0)	0.070
Dental	104	88.5 (82.0–94.3)	86.5 (79.6–92.8)	81.7 (73.8–89.2)	79.8 (71.8–87.0)	77.9 (69.7–85.6)	0.204

Values are given as sensitivity (95% CI). Confidence intervals were calculated using the patient-level cluster bootstrap. *p*-values were obtained from omnibus Wald chi-square tests for the overall reader effect in GEE models accounting for patient-level clustering. LLM 1–3 are defined in Section 2.10.

**Table 5:** Finding-level sensitivity, cluster-robust 95% confidence intervals, and Cochran’s Q results.

Finding	*n*	Expert 1	Expert 2	LLM 1	LLM 2	LLM 3	Cochran Q	q
Tonsillolith	46	95.7 (88.7–100.0)	93.5 (85.5–100.0)	91.3 (82.5–98.0)	87.0 (76.3–95.9)	84.8 (73.3–94.3)	5.06	0.506
Mild mucous retention pseudocyst	56	94.6 (88.1–100.0)	92.9 (85.5–98.4)	89.3 (81.0–96.5)	85.7 (75.8–94.3)	82.1 (71.4–91.8)	6.19	0.417
Enostosis	55	92.7 (85.1–98.3)	90.9 (82.5–98.0)	87.3 (77.8–95.6)	85.5 (75.5–94.2)	81.8 (71.0–91.5)	3.86	0.637
Residual root	50	90.0 (81.0–97.8)	88.0 (78.4–96.1)	82.0 (70.7–92.2)	80.0 (68.2–90.7)	80.0 (68.1–90.5)	3.19	0.677
Condensing osteitis	54	87.0 (77.6–95.4)	85.2 (75.0–94.2)	81.5 (70.1–91.6)	79.6 (67.9–89.5)	75.9 (64.4–87.5)	2.65	0.695
Sialolith	45	84.4 (73.5–94.6)	82.2 (70.3–92.9)	80.0 (67.6–91.2)	80.0 (67.5–91.2)	77.8 (64.9–89.7)	0.72	0.949
Carotid artery calcification	37	91.9 ^a^ (81.8–100.0)	89.2 ^a^ (78.4–97.5)	67.6 ^ab^ (51.5–82.1)	54.1 ^b^ (37.5–70.2)	45.9 ^b^ (29.6–62.2)	27.81	<0.001
Extensive maxillary sinus pathology	55	92.7 ^a^ (85.2–98.4)	90.9 ^a^ (82.5–98.0)	78.2 ^ab^ (66.7–88.6)	72.7 ^ab^ (60.9–84.2)	65.5 ^b^ (52.5–78.0)	18.24	0.005
Large osseous lesion	40	95.0 (87.5–100.0)	92.5 (83.3–100.0)	87.5 (75.8–97.1)	82.5 (70.2–93.5)	75.0 (60.5–87.5)	9.36	0.158

Values are given as finding-specific sensitivity (95% CI). Confidence intervals were calculated using the patient-level cluster bootstrap. q-values were obtained by applying the Benjamini–Hochberg correction to the nine Cochran’s Q tests. For the two findings that remained significant after correction, values that do not share a superscript letter differ significantly in Bonferroni-adjusted pairwise exact McNemar comparisons (adjustment for 10 reader pairs within each finding). LLM 1–3 are defined in Section 2.10.

## Data Availability

Most of the data generated or analyzed are included in the article. The remaining datasets used and/or analyzed during the current study are available from the corresponding author upon request.

## Supplementary Materials

The following supporting information can be downloaded at: https://www.mdpi.com/article/10.3390/healthcare14182948/s1, File S1: STARD 2015 checklist; File S2: CLAIM 2024 update checklist; Table S1: Bonferroni-adjusted pairwise comparisons among the three large language models (LLMs) for overall sensitivity, specificity, and accuracy, derived from the generalized estimating equation (GEE) models. References [39,40] are cited in the Supplementary Materials.

## Abbreviations

The following abbreviations are used in this manuscript:

CBCT	Cone-beam computed tomography
LLM	Large language model
GEE	Generalized estimating equations
FDR	False discovery rate
STARD	Standards for Reporting Diagnostic Accuracy Studies
CLAIM	Checklist for Artificial Intelligence in Medical Imaging
FOV	Field of view
API	Application programming interface
FNR	False-negative rate
FPR	False-positive rate
CI	Confidence interval
OR	Odds ratio
