# Supplementary material for: Comparison of Multimodal Large Language Models and Oral and Maxillofacial Radiologists in the Detection of Incidental Findings on Panoramic Radiographs: A CBCT-Referenced Diagnostic Accuracy Study
Source: Healthcare (Basel). 2026 Sep 10;14(18):2948. doi: 10.3390/healthcare14182948 (PMC13606792; doi:10.3390/healthcare14182948)
Supplement: Supplementary file 1 [file healthcare-14-02948-s001.zip › healthcare-4541220-supplementary.pdf]

File S1. STARD 2015 CHECKLIST

Standards for Reporting Diagnostic Accuracy Studies

Manuscript Title: Comparison of Large Language Models and Oral and Maxillofacial Radiologists in the Detection of Incidental Findings on Panoramic Radiographs: A CBCT-Referenced Diagnostic Accuracy Study

Source: Bossuyt PM, Reitsma JB, Bruns DE, et al. STARD 2015: an updated list of essential items for reporting diagnostic accuracy studies. BMJ 2015;351:h5527.

No	Item	Status	Page / Section and Description
**1**	Identification as a study of diagnostic accuracy using at least one measure of accuracy (such as sensitivity, specificity, predictive values, or AUC)	Yes	Title states: "…A CBCT-Referenced Diagnostic Accuracy Study."
**2**	Structured summary of study design, methods, results, and conclusions	Yes	Abstract is structured under Background/Objectives, Methods, Results, and Conclusions.
**3**	Scientific and clinical background, including the intended use and clinical role of the index test	Yes	Introduction.
**4**	Study objectives and hypotheses	Partially	The aim is stated in the final paragraph of the Introduction; an explicit a priori hypothesis statement is not included.
**5**	Whether data collection was planned before the index test and reference standard were performed (prospective) or after (retrospective)	Yes	Materials and Methods, Study Design.
**6**	Eligibility criteria	Yes	Materials and Methods, Sample Selection and Eligibility Criteria.
**7**	On what basis potentially eligible participants were identified	Yes	Materials and Methods, Data Source and Image Archive.
**8**	Where and when potentially eligible participants were identified (setting, location, and dates)	Yes	University Department of Oral and Maxillofacial Radiology; January 2021-December 2025.
**9**	Whether participants formed a consecutive, random, or convenience series	Yes	States that a non-consecutive, finding-based stratified sampling approach was used.
**10a**	Index test, in sufficient detail to allow replication	Yes	Expert and LLM evaluation protocols (prompt text, image preparation, access dates).
**10b**	Reference standard, in sufficient detail to allow replication	Yes	CBCT reference evaluation protocol (three radiologists, structured form, consensus process).
**11**	Rationale for choosing the reference standard (if alternatives exist)	Partially	States indirectly that CBCT overcomes the two-

No	Item	Status	Page / Section and Description
			dimensional limitations of panoramic radiography; no direct comparative rationale against alternatives (e.g., histopathology) is given.
**12a**	Definition of and rationale for test positivity cut-offs or result categories of the index test, distinguishing pre-specified from exploratory	Yes	The binary "Present/Absent" format was pre-specified via the standardized prompt.
**12b**	Definition of and rationale for test positivity cut-offs or result categories of the reference standard	Yes	Materials and Methods, Table 1.
**13a**	Whether clinical information and reference standard results were available to the performers/readers of the index test	Yes	States that expert and LLM readers were blinded.
**13b**	Whether clinical information and index test results were available to the assessors of the reference standard	Yes	States that reference readers were blinded to panoramic images and index test results.
**14**	Methods for estimating or comparing measures of diagnostic accuracy	Yes	Statistical Analysis: GEE, cluster bootstrap, Cochran's Q, Benjamini–Hochberg correction.
**15**	How indeterminate index test or reference standard results were handled	Yes	States that no indeterminate/invalid responses occurred; reference standard disagreements were resolved by consensus.
**16**	How missing data on the index test and reference standard were handled	Partially	The absence of missing data can be inferred from the reported counts; no explicit confirmatory statement is included.
**17**	Any analyses of variability in diagnostic accuracy, distinguishing pre-specified from exploratory	Yes	Risk- and category-based subgroup analyses are labeled as secondary/exploratory.
**18**	Intended sample size and how it was determined	No	Not reported.
**19**	Flow of participants, using a diagram	Partially	Presented in Figure 1; the number of cases excluded from the initial candidate archive pool is not separately itemized in the diagram.
**20**	Baseline demographic and clinical characteristics of participants	No	Not reported.
**21a**	Distribution of severity of disease in those with the target condition	Partially	Operational criteria are presented in Table 1; the size/extent distribution per finding is not separately reported in Results.

No	Item	Status	Page / Section and Description
**21b**	Distribution of alternative diagnoses in those without the target condition	Partially	Additional findings beyond the nine predefined findings are not reported.
**22**	Time interval and any clinical interventions between index test and reference standard	Yes	States that panoramic radiography and CBCT were performed on the same day.
**23**	Cross tabulation of the index test results by the results of the reference standard	Partially	Sensitivity/specificity estimates are presented in Tables 2-5; raw TP/FP/FN/TN counts are not shown in a separate cross-tabulation.
**24**	Estimates of diagnostic accuracy and their precision (such as 95% confidence intervals)	Yes	Tables 2-5.
**25**	Any adverse events from performing the index test or the reference standard	Not applicable	Image-interpretation study; no risk of adverse events.
**26**	Study limitations, including sources of potential bias, statistical uncertainty, and generalizability	Yes	Limitations section.
**27**	Implications for practice, including the intended use and clinical role of the index test	Yes	Discussion and Conclusion.
**28**	Registration number and name of registry	No	The study was not prospectively registered.
**29**	Where the full study protocol can be accessed	No	A separate protocol has not been published.
**30**	Sources of funding and other support; role of funders	Yes	Funding statement (back matter): "This research received no external funding."

File S2. CLAIM 2024 UPDATE CHECKLIST

Checklist for Artificial Intelligence in Medical Imaging

Manuscript Title: Comparison of Large Language Models and Oral and Maxillofacial Radiologists in the Detection of Incidental Findings on Panoramic Radiographs: A CBCT-Referenced Diagnostic Accuracy Study

Source: Tejani AS, Klontzas ME, Gatti AA, et al. Checklist for Artificial Intelligence in Medical Imaging (CLAIM): 2024 Update. Radiol Artif Intell 2024;6(4):e240300.

This study is a zero-shot diagnostic performance evaluation of pre-trained commercial large language models and does not involve the development or training of a new model; items related to model training and data partitioning (19-20, 22-27, 31-32, 34) are therefore marked "Not applicable."

No	Item	Status	Page / Section and Description
**1**	Identification as a study of AI methodology, specifying the category of technology used (e.g., deep learning)	Yes	Title and Abstract state "large language models."

No	Item	Status	Page / Section and Description
**2**	Summary of study design, methods, results, and conclusions	Yes	Abstract.
**3**	Scientific and/or clinical background, including the intended use and role of the AI approach	Yes	Introduction.
**4**	Study aims, objectives, and hypotheses	Partially	The aim is stated in the final paragraph of the Introduction; an explicit hypothesis statement is not included.
**5**	Prospective or retrospective study	Yes	Materials and Methods, Study Design.
**6**	Study goal	Partially	It can be inferred from Materials and Methods that this is a comparative evaluation of pre-trained commercial models; a separate classifying statement is not included.
**7**	Data sources	Yes	Materials and Methods, Data Source and Image Archive.
**8**	Inclusion and exclusion criteria	Yes	Materials and Methods, Sample Selection and Eligibility Criteria.
**9**	Data preprocessing	Yes	JPEG export, resizing to 1536×832 pixels, and compression level are described.
**10**	Selection of data subsets	Yes	Finding-based stratified sampling process.
**11**	De-identification	Partially	States that patient name/ID number were removed; reference to a specific regulatory framework (e.g., GDPR/HIPAA) is not included.
**12**	Missing data	Partially	See STARD item 16.
**13**	Image acquisition protocol	Yes	Device model, kVp, FOV options, and voxel size are specified.
**14**	Definition of method(s) used to obtain the reference standard	Yes	Table 1 and the CBCT reference protocol.
**15**	Rationale for choosing the reference standard	Partially	See STARD item 11.
**16**	Source of reference standard annotations	Yes	Three radiologists; years of experience specified.
**17**	Annotation of test set	Partially	A structured form is mentioned; specific software name is not given.

No	Item	Status	Page / Section and Description
**18**	Measures of inter- and intra-rater variability	Partially	Interobserver variability is reported (Cohen's kappa for the two experts; Fleiss' kappa for the three reference radiologists). Intraobserver (intra-rater) variability was not assessed, as each reader evaluated each image once.
**19**	How data were assigned to partitions	Not applicable	No model training was performed; pre-trained commercial models were evaluated zero-shot.
**20**	Level at which partitions are disjoint	Not applicable	Same rationale as item 19.
**21**	Intended sample size	No	Not reported.
**22**	Detailed description of model	Not applicable	Evaluated models are closed-source commercial systems.
**23**	Software libraries, frameworks, and packages	Not applicable	No model training was performed (see item 29 for statistical software).
**24**	Initialization of model parameters	Not applicable	Same rationale as item 19.
**25**	Details of training approach	Not applicable	Same rationale as item 19.
**26**	Method of selecting the final model	Not applicable	Same rationale as item 19.
**27**	Ensembling technique	Not applicable	Same rationale as item 19.
**28**	Metrics of model performance	Yes	Sensitivity, specificity, accuracy, false negative/positive rates.
**29**	Statistical measures of significance and uncertainty; statistical software and version	Yes	GEE, bootstrap, Cochran's Q, FDR correction; IBM SPSS Statistics 26.0 and Python 3.12.13 specified.
**30**	Robustness or sensitivity analysis	Partially	A separate robustness analysis is not presented.
**31**	Methods for explainability or interpretability	Not applicable	Commercial models are closed-source; the consumer interface does not provide explainability output.
**32**	Evaluation on internal data	Not applicable	No training/internal-test split exists.

No	Item	Status	Page / Section and Description
**33**	Testing on external data	No	Single-center design; noted in the Limitations section.
**34**	Clinical trial registration	Not applicable	Retrospective diagnostic accuracy study; clinical trial registration is not required.
**35**	Numbers of patients or examinations included and excluded	Partially	See STARD item 19.
**36**	Demographic and clinical characteristics of cases in each partition and dataset	No	Not reported.
**37**	Performance metrics and measures of statistical uncertainty	Yes	Consensus process is described; risk/category/finding subgroups are reported.
**38**	Estimates of diagnostic performance and their precision; class imbalance	Yes	Sensitivity and false negative rate are reported alongside accuracy.
**39**	Failure analysis of incorrect results	Partially	Representative cases are presented in Figure 3; a formal confusion matrix is not included.
**40**	Study limitations	Yes	Limitations section.
**41**	Implications for practice, including intended use and/or clinical role	Yes	Discussion and Conclusion.
**42**	Reference to the full study protocol or additional technical details	No	A separate protocol has not been published.
**43**	Statement about the availability of software, trained model, and/or data	Yes	Data Availability Statement (back matter): data included in the article; remaining datasets available from the corresponding author upon request. A statement on software/trained-model availability is not applicable, as only closed-source commercial models were evaluated.
**44**	Sources of funding and other support; role of funders	Yes	Funding statement (back matter): "This research received no external funding."

Supplementary Material

Comparison of Multimodal Large Language Models and Oral and Maxillofacial Radiologists in the Detection of Incidental Findings on Panoramic Radiographs: A CBCT-Referenced Diagnostic Accuracy Study

İsmail Çapar, Utku Cem Hasırcı, Didem Dumanlı Kusay, Edanur Altın, Gediz Geduk

Table S1. Bonferroni-adjusted pairwise comparisons among the three large language models (LLMs) for overall sensitivity, specificity, and accuracy, derived from the generalized estimating equation (GEE) models.

Metric	Comparison	OR (unadjusted 95% CI)	Adjusted p	Result
Sensitivity	LLM 1 vs. LLM 2	1.30 (0.92–1.84)	1.000	Not significant
	LLM 1 vs. LLM 3	1.65 (1.17–2.32)	0.039	Significant
	LLM 2 vs. LLM 3	1.27 (0.93–1.74)	1.000	Not significant
Specificity	LLM 1 vs. LLM 2	0.90 (0.78–1.03)	1.000	Not significant
	LLM 1 vs. LLM 3	1.10 (0.96–1.27)	1.000	Not significant
	LLM 2 vs. LLM 3	1.23 (1.06–1.42)	0.062	Not significant
Accuracy	LLM 1 vs. LLM 2	0.95 (0.84–1.08)	1.000	Not significant
	LLM 1 vs. LLM 3	1.17 (1.03–1.32)	0.158	Not significant
	LLM 2 vs. LLM 3	1.23 (1.08–1.40)	0.022	Significant

OR, odds ratio; CI, confidence interval. ORs compare the odds of correct classification for the first-named model with those for the second and were estimated from the GEE models described in Section 2.18 (n = 438 reference-positive, 4,062 reference-negative and 4,500 total observations for the sensitivity, specificity and accuracy models, respectively). Confidence intervals are unadjusted for multiplicity, whereas p-values are Bonferroni-adjusted for the 10 pairwise reader comparisons within each metric (capped at 1.000). An interval excluding 1 may therefore accompany a non-significant adjusted p-value, as for specificity (LLM 2 vs. LLM 3) and accuracy (LLM 1 vs. LLM 3); in these cases, the adjusted p-value governs the inference and the interval should be read as descriptive. LLM 1, Claude Sonnet 4.6; LLM 2, Gemini 3; LLM 3, ChatGPT-5.3. Expert–LLM and expert–expert comparisons are given as superscript letters in Table 2 of the main text.
